# The Role of Difficulty in Identifying and Describing Feelings in Non-Suicidal Self-Injury Behavior (NSSI): Associations With Perceived Attachment Quality, Stressful Life Events, and Suicidal Ideation

**DOI:** 10.3389/fpsyg.2018.00318

**Published:** 2018-03-13

**Authors:** Rita Cerutti, Antonio Zuffianò, Valentina Spensieri

**Affiliations:** ^1^Department of Dynamic and Clinic Psychology, Sapienza University of Rome, Rome, Italy; ^2^Department of Psychology, Liverpool Hope University, Liverpool, United Kingdom

**Keywords:** alexithymic features, NSSI, attachment, stressful life events, adolescents

## Abstract

**Objective:** Core alexithymic features, such as the difficulty in identifying and describing feelings, are associated with poor attachment styles and emotional trauma, which influence the capacity to regulate affect. Additionally, emotional regulation has been found to be the most commonly identified function associated with non-suicidal self-injury behavior (NSSI) in adolescents as they attempt to modulate strong emotions. However, few studies have examined the link between difficulty in identifying and describing feelings (core components of alexithymia), NSSI behaviors, quality of attachment, life stressors and suicidal ideation in healthy early adolescents. Consequently, this study aims to investigate these constructs and the relationship among them in a large non-clinical sample of adolescents.

**Methods:** Seven hundred and nine middle school students (50.4% males), aged 10–15 years (*M* = 12.6; *SD* = 1.06) were involved in this study. In order to investigate the variables considered in the study, the following measures were administered: the Deliberate Self-Harm Inventory exploring non-suicidal self-injurious behaviors; the Alexithymia Questionnaire for Children examining difficulty in identifying and describing feelings; the Inventory of Parent and Peer Attachment assessing the quality of parental and peer attachment; the Life Stressor Checklist-Revised outlining stressful/traumatic events and the Children’s Depression Inventory evaluating suicidal ideation.

**Results:** We found significantly positive relationships among difficulty in identifying and describing feelings, NSSI behaviors, stressful events, and suicidal ideation. Data indicated a significant negative association of difficulty in identifying and describing feelings with quality of attachment to parents and peers. Further findings highlighted that difficulty in identifying and describing feelings significantly mediated the effect of quality of attachment (parent and peer) on NSSI and suicidal ideation.

**Conclusion:** The ability to identify and describing feelings is important to managing emotional expression and understanding the feelings of others, both crucial in attaining successful interpersonal relationships. Our data revealed that, while controlling for stressful life events, low levels of attachment may increase adolescents’ difficulty in identifying and describing their own feelings, which in turn may increase the risk of both NSSI and suicidal ideation.

## Introduction

In recent years, there has been growing interest in research on alexithymia, as documented by a series of major systematic reviews in the international literature. Although the literature is not always unanimous about the definition of “alexithymia,” research suggests that alexithymia is a multi-dimensional deficit in affect recognition and regulation (Timoney and Holder, 2013). For some authors, it refers to a personality construct normally distributed in the population (Parker et al., 2008) whilst others use the term to denote a limited ability to identify, describe and communicate one’s feelings, which in turn reflects difficulties in affective self-regulation (Taylor et al., 1997). Thus a reduced ability to connect emotions with words (or “absence of words for emotions” as attested by its etymology from the Greek alpha = absence, lexis = language, thymos = emotions) has been used to define the construct of alexithymia (Taylor et al., 1997; Taylor, 2010). In general, the following clinical features have been considered as dimensions of alexithymia: difficulty in identifying and describing emotions, difficulty in distinguishing between subjective emotional states and the somatic components of emotional activation, poverty of imaginative processes, and cognitive style oriented toward external reality.

In line with previous investigation of healthy adults (Paivio and McCulloch, 2004), difficulties in identifying and describing inner feelings have been conceptualized as core components of alexithymia which may be associated with problems in building and maintaining close relationships with others and using social support to protect themselves against the potentially pathological influences of stressful events (Kojima et al., 2003). The difficulty in “identifying and describing ones’ own inner feelings” may make people reluctant to participate in social activities (Kojima, 2012).

It has been suggested that alexithymia is broadly associated with various mental and physical health problems (Cerutti et al., 2016, 2017; Gatta et al., 2016). Individuals with alexithymia are more likely to have a limited ability to adaptively cope with stressful conditions and tend to encompass unhealthy behaviors such as alcohol and drug use (Lumley et al., 2007). Some studies have indicated that self-injuries are associated with a higher level of alexithymia than non-self-injuries among high school students and adolescent inpatient populations (Garisch and Wilson, 2010; Cerutti et al., 2014). Other studies have highlighted that adolescents inpatients with non-suicidal self-injury (NSSI) are more likely to have a pervasive and comprehensive Theory of Mind impairment (Laghi et al., 2016).

Non-suicidal self-injury is defined as the intentional injuring of one’s body without apparent suicidal intent and for reasons not socially acceptable within one’s culture (Muehlenkamp et al., 2012). Similar to other risky behaviors (e.g., alcohol and substance abuse), it typically begins between the age of 13 and 15 years (Favazza and Rosenthal, 1993; Hilt et al., 2008; Cerutti et al., 2011; Muehlenkamp et al., 2012). The transition to adolescence seems to mark a developmental period distinguished by its increased rates of NSSI behaviors (Peterson et al., 2008; Cerutti et al., 2014; Manca et al., 2014). International studies have revealed a wide variability in prevalence rates, with percentages ranging from 12 to 56% in non-clinical populations of adolescents (Cerutti et al., 2011; Muehlenkamp et al., 2012) and from 20 to 80% in clinical populations (Hilt et al., 2008; Ferrara et al., 2012; Cerutti et al., 2014; Kara et al., 2015).

During the last two decades, NSSI has attracted the attention of researchers among clinical and non-clinical settings (Klonsky and Muehlenkamp, 2007; Nixon et al., 2008; Nixon and Heath, 2009; Muehlenkamp et al., 2012; International Society for the Study of Self-Injury, 2015; Zetterqvist, 2017) since this phenomenon is widespread across the Western world. It represents a serious public health problem (Klonsky et al., 2013; Cerutti et al., 2014; Serafini et al., 2017) owing to the greater risk for later suicidal behavior especially among individuals who engaging NSSI repeatedly (Joiner, 2005; Joiner et al., 2012; Grandclerc et al., 2016).

There is evidence that NSSI is generally used to cope with distressing negative affective states, especially anger and depression, and mixed emotional states. However, to date, there is a paucity of studies exploring how individuals who engage in self-injury may experience difficulty expressing and verbalizing emotions in early adolescence and adolescence (Cerutti et al., 2014). Research is needed to examine a broader range of stressful childhood experiences, as few studies have examined the relationship between negative experiences or stressful life events, alexithymia and NSSI among young people. In a previous study, Paivio and McCulloch (2004) found that alexithymia partially mediated the relationship between traumatic experience and self-injurious behaviors among female undergraduate students.

Furthermore, it has been suggested that stressful and traumatic life events (e.g., abuse) in childhood may be related to self-injurious behavior in adulthood (Ross and McKay, 1979; Terr, 1991; van der Kolk et al., 1991). Studies have also highlighted that not all individuals with a history of abuse later engage in NSSI, and not all individuals who self-injure have been abused (Klonsky and Muehlenkamp, 2007). The stress exposure model of psychopathology indicate that experiencing higher rates of life stressors or negative life events contribute to a higher risk for negative mental health outcomes (Liu et al., 2014). Nock (2010) suggested that stressful life events have the similarly prominent role as proximal risk factors for NSSI behavior, since in presence of stressful life events specific physiological responses are experienced by some individuals, who further may be at risk for engaging in NSSI as a coping strategy. This is more evident in individuals with difficulties in emotional regulation (Tang et al., 2016). Additionally, negative life events ranging from traumatic stressors to major life changes are consistently associated with suicidal ideation (Liu et al., 2014). It is well known that environmental influences have a particular impact on children’s psychological development, especially the ‘emotional climate’ provided by the parents (Taylor, 2010). Studies have highlighted that early attachment difficulties may contribute to later self-injurious behaviors (van der Kolk et al., 1996; Conterio and Lader, 1998; Walsh, 2006). Specifically, Gratz et al. (2002) examined the role of the parent–child relationship as a risk factor for NSSI revealing that emotional neglect and the quality of the parent–child relationship were associated with risk of developing NSSI later. In view of new forms of sociality, adolescents make an important transition from family to peer group through gradual autonomy from primary attachment figures. The interest toward the peer group, with whom adolescents can share experiences and affection, becomes a determining factor to promote the growth and construction of personal identity.

Although research supports the relation between stressful life events and NSSI behaviors, to date there are few studies that have been undertaken on the degree to which stressful life events lead to NSSI among adolescents, except for research focusing on childhood abuse and other severe early life adversities (Tang et al., 2016). Only a small number of empirical studies have investigated the relationship between NSSI and core alexithymic features (i.e., difficulty in identifying and describing feelings) in different populations while no systematic review of the literature has been conducted to date. Moreover, the overall relationships among the difficulty in identifying and describing feelings, quality of parental and peer attachment, NSSI, suicidal ideation, and of stressful life events among young adolescents have not been sufficiently investigated.

### The Present Study

In light of the above considerations, this study aimed to investigate the plausibility of a theoretical model in which low quality of attachment toward both peers and parents could represent emotional risk factors that may predispose adolescents to have increased difficulties in identifying and describing their own feelings which, in turn, may heighten the likelihood of developing both NSSI and suicidal ideation. Specifically, we hypothesized NSSI and suicidal ideation to be positively correlated with adolescents’ difficulty in identifying and describing feelings. NSSI, suicidal ideation and difficulty in identifying and describing feelings, instead, were hypothesized to be negatively related to the quality of attachment toward both parents and peers. Lastly, in line with previous arguments (Klonsky and Muehlenkamp, 2007; Cerutti et al., 2011; Laukkanen et al., 2013) highlighting the positive relationship of stressful life events to NSSI, suicidal ideation, and difficulty in identifying and describing feelings, we also included in our model the number of stressful life events as a control variable.

## Materials and Methods

### Participants

Seven hundred and nine Italian early adolescents (50.4% male), aged 10–15 years were involved in the present study. Participants were recruited in two middle schools in Rome. Exclusion criteria for participation included the presence of a diagnosed psychiatric illness and/or history of psychiatric treatment, history of significant neurological illness or brain injury, history of chronic pains and recurrent somatic symptoms. The vast majority (92.5%) of the participants were Caucasian.

### Procedure

The participants and their parents/caregivers gave their written informed consent before inclusion in the present study. The administration of the self-reported questionnaires took place during school time in the classrooms. Anonymity of participants was ensured. Questionnaires took approximately 30–45 min to complete. All participants completed the questionnaire battery.

This study was approved by the Ethics Committee of the Department of Dynamic and Clinical Psychology, Sapienza University of Rome.

### Measures

#### Deliberate Self-Harm Inventory

The Deliberate Self-Harm Inventory (DSHI; Gratz, 2001) was used to assess non-suicidal self-injury behavior (NSSI). The DSHI is a 17-item self-report measure that assesses lifetime history of NSSI (defined as the deliberate, direct destruction of body tissue without suicidal intent), including frequency, duration, and type of NSSI behavior. The DSHI was recently validated in the Italian context by Cerutti et al. (2012) and was found to have adequate internal consistency, and good convergent and discriminant validity. An overall score of NSSI was created by summing participants’ scores on the 10 items (Gratz, 2006). In the present study, good reliability (α = 0.62) was found.

#### Suicidal Ideation

Suicidal ideation was assessed by using one item from the Children’s Depression Inventory-2 (CDI-2; Kovacs, 2015; Italian adaptation by Camuffo and Cerutti, in press) The item has three response options that score 0 (*absence of suicidal ideation*), 1 (*mild suicidal ideation*), or 2 (*severe suicidal ideation*). For our purposes, we recoded this item by merging the last two response-choices into one. The new dichotomous variable ranged from 0 (absence of suicidal ideation) and 1 (presence of suicidal ideation).

#### Difficulty in Identifying and Describing Feelings

For this study, we assessed the difficulty in identifying feelings (DIF) and describing feelings (DDF) by using the difficulty in identifying feelings subscale (DIF-S) and difficulty in describing feelings subscale (DDS) of the Alexithymia Questionnaire for Children (AQC; Rieffe et al., 2006; Di Trani et al., 2009). The DIF-S is composed by seven items (e.g., “I am often confused about the way I am feeling inside”; α = 0.77) whereas the DDF-s by five items (e.g., “I find it difficult to say how I feel inside; α = 0.64). Both subscales are scored on a three-point rating scale (from 0 = not true to 2 = often true) and assessed the degree to which children feel unable to recognize and describe their own feelings. Both subscales showed positive correlations with somatic problems and several negative moods like anger, sadness, and fear (Rieffe et al., 2006).^1^

#### The Inventory of Parent and Peer Attachment

The Inventory of Parent and Peer Attachment (IPPA; Armsden and Greenberg, 1987) was used to measure the quality of parent (IPPA-PA) and peer (IPPA-PE) attachment in adolescence. The good psychometric properties of the IPPA have been already confirmed in several studies with Italian samples (Laghi et al., 2009; Pace et al., 2011). The Parent Attachment Scale consists of 28 items whereas the Peer Attachment Scale consists of 25 items. The items of both instruments were scored on a five-point scale (from 1 = “not true at all” to 5 = “completely true”) and assessed three dimensions of attachment, respectively: “Trust,” “Communication,” and “Alienation.” The Trust scale measures the extent to which adolescents trust their parents (α = 0.82) and peers (α = 0.88) to respect and accept their feelings (e.g., “My parents/peer respect my feelings”). The Communication scale measures the extent adolescents experience having a high quality of communication (e.g., “When my parents/friends know that something is bothering me, they ask me about it”) with their parents (α = 0.78) and peers (α = 0.85). The Alienation scale measures the degree to which adolescents experience negative feelings (e.g., “I don’t get much attention from my parents/friends”) toward parents (α = 0.80) and peers (α = 0.71). For our purposes, the individual’s mean score on the three scales was considered as the indicator of parent and peer attachment. Furthermore, these aggregated scores were highly reliable: alphas for the global attachment scale toward parent and peers were, respectively, 0.84 and 0.77.^2^

#### Life Stressor Checklist-Revised

A reduced 13-item version of the *Life Stressor Checklist-Revised* (Wolfe et al., 1996; Giannantonio, 2005) was used to assess the presence and impact of a variety of stressful or traumatic events that may have occurred in the participant’s life. Specifically, participants are asked to indicate whether or not they experienced each event, as well as their level of distress in response to the events they endorsed. For the present study, the dichotomous responses (yes vs. no) to all of the items were summed to create an overall measure of the number of stressful life events experienced.

### Data Analytical Approach

**FIGURE 1:**
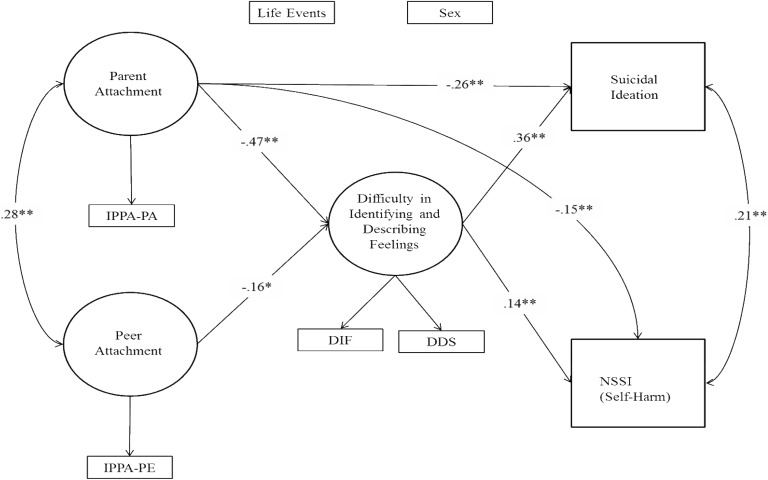
Mediational model. Variance explained (*R*^2^). Difficulty in identifying and describing feelings (*R*^2^ = 0.434); suicidal ideation (*R*^2^ = 0.328); NSSI (*R*^2^ = 0.156). The effects of sex and stressful events were estimated but not depicted for the sake of simplicity.

As a preliminary step, we computed the correlations among the variables of interest. Then, we examined the hypothesized model (**Figure 1**) in a structural equation modeling (SEM) framework using M*plus*8 (Muthén and Muthén, 1998–2017). A general latent factor measuring participants’ difficulty in identifying and describing their feelings was modeled by using the two subscales DIF-S and DDS-S. The composite mean scores of IPPA-PA and IPPA-PE scales were used as the indicators to model parental and peer attachment. These variables were posited as single indicator latent variables by estimating the error terms from their reliabilities (Kline, 2010). As suicidal ideation was coded as a dichotomous variable, parameter estimates were based on the Weighted Least Squares Mean-Variance adjusted (WLSMV) estimator. This method is particularly suited for dealing with categorical data. Specifically, M*plus* 8 computed probit regression coefficients (Muthén and Muthén, 1998–2017) to assess the impact of our predictors on the dichotomous outcome variable suicidal ideation (Muthén and Muthén, 1998–2017). Non-significant χ^2^ likelihood ratio statistic, comparative fit index (CFI) and Tucker-Lewis index (TLI) greater than 0.95, and root mean square error of approximation (RMSEA) values lesser than 0.05 (Kline, 2010) were considered as indicators of a good model fit. According to the principle of *parsimony* (i.e., reducing the model’s complexity by increasing the number of degrees of freedom without worsening the fit), we tested a series of increasingly liberal mediational models, in which direct paths from independent variables to our outcome variables (i.e., suicidal ideation and self-harm) were sequentially freely estimated. In detail, we estimated a full mediational model (i.e., without direct effects from our focal predictors parent and peer attachment to our distal outcomes suicidal ideation and self-harm). Next, we added the direct effects to evaluate if a partial mediational model significantly fit the data. According to MacKinnon (2008), paths were retained only if they resulted in a significant increment of model fit (we compared these nested models by using the DIFFTEST function in M*plus*8; Muthén and Muthén, 1998–2017). In line with recent recommendations (Hayes and Scharkow, 2013), we computed the 95% bias-corrected confidence intervals (CI) to formally test the significance of our hypothesized mediational effects (ab). If the lower and upper limits of the 95% CI did not include zero, we concluded that the mediated effect was statistically different from zero. Finally, both participants’ sex and life events were used as control variables in order to partial out their effects.

## Results

### Sample Characteristics

The participants mean age was 12.6, with a standard deviation of 1.06. Among adolescents 83% reported at least one brother or sister. Socio-demographic characteristics of parents are described in **Table 1**.

**Table 1:** Sample description.

		Total (*N* = 790)%	Boys (*n* = 357)%	Girls (*n* = 352)%
Educational	Primary	20		
level	Secondary	42		
	University	33		
	Missing	5		
Civil status	Single	0.7		
	Married/cohabiting	77.2		
	Separated/divorced	12.4		
	Widowed	2.3		
	Missing	7.4		
NSSI behavior	Presence	28.8	16.1	12.7
	Absence	71.2	34.3	36.9


According to Gratz inventory (Gratz, 2001), results indicated that 204 adolescents (28.8%) endorsed at least one lifetime episode of NSSI and 97 of them (13.7%) reported more than one episode with at least two different methods while only1.4% reported engaging in repetitive NSSI (≥5 episodes) during the last year. Detailed results are reported in **Table 1**. No statistical differences between boys and girls emerged regarding the presence or absence of NSSI behavior.

### Correlation Analysis

Correlations were mostly as expected (**Table 2**). Suicidal ideation, NSSI, difficulty in identifying and describing feelings were (a) positively correlated with stressful life events and (b) negatively correlated with both parental and peer attachment. Girls were more likely to report higher difficulties in identifying their feelings than boys.

**Table 2:** Correlation matrix.

	NSSI	Sui-Id	DIF	DDF	IPPA-PA	ATTP	Str. events	Sex
NSSI	—	0.320***	0.261***	0.212***	-0.276***	-0.131***	0.314***	-0.064
Sui-Id		-	0.357***	0.232***	-0.331***	-0.154***	0.227***	0.010
DIF			-	0.603***	-0.451***	-0.164***	0.351***	0.096*
DDF				-	-0.391***	-0.219***	0.284***	0.060
IPPA-PA					-	0.232***	-0.316***	0.008**
IPPA-PE						-	-0.129***	0.243***
Str. events							-	-0.037
Sex								-


*NSSI, non-suicidal self-injury; Sui-Id, suicidal ideation (0 = absence of suicidal ideation, 1 = presence of suicidal ideation); DIF, difficulty in identifying feelings; DDF, difficulty in describing feelings; IPPA-PA, parent attachment; IPPA-PE, peer attachment; sex (0 = boys, 1 = girls). ^∗^*p* < 0.05, ^∗∗^*p* < 0.01, ^∗∗∗^*p* < 0.001.*

### Mediational Models

The full mediational model showed a marginal fit χ^2^(9) = 25.82, *p* < 0.001, CFI = 0.98, TLI = 0.94, RMSEA = 0.05 [90% CI: 0.03, 0.08]. Thus, we proceeded by testing two partial mediational models. First, we added the direct paths from peer attachment to both suicidal ideation and NSSI. This partial mediational model showed a marginal fit χ^2^(7) = 26.01, *p* < 0.001, CFI = 0.98, TLI = 0.92, RMSEA = 0.06 [90% CI: 0.04, 0.09] and was not statistically different from the full mediational model Δχ^2^(2) = 2.22, *p* = 0.33. Then, we estimated a second partial mediational model in which we added the direct paths from parental attachment to both suicidal ideation and NSSI. This partial mediational model showed a perfect fit to the data and χ^2^(7) = 10.01, *p* = 0.19, CFI = 1.00, TLI = 0.99, RMSEA = 0.03 [90% CI: 0.00, 0.06] and was statistically different from the full model Δχ^2^(2) = 13.91, *p* = 0.001, thereby providing evidence for freely estimating the direct effects of parental attachment.

Once selected the second partial mediational model as the best fitting one, we computed the 95% CI for our hypothesized mediational effects. Results indicated that higher levels of parental attachment (*ab* = -0.27, [95% CI: -0.44, -0.15]) and peer attachment (*ab* = -0.10, [95% CI: -0.21, -0.03]) were related to a lack of suicidal ideation via the mediational role of difficulty in identifying and describing feelings (the 95% asymmetric lower and upper CI limits did not include zero). Similarly, the difficulty in identifying and describing feelings significantly mediated the effect of parental attachment (*ab* = -0.18, [95% CI: -0.23, -0.02]) and peer attachment (*ab* = -0.04, [95% CI: -0.10, -0.01]) on NSSI.

### Alternative Mediational Model

We also investigated the fit of three alternative models representing plausible alternative explanations of the covariance structure. First, we tested an alternative model in which we posited NSSI and suicidal ideation as independent variables and parental and peer attachment as distal outcomes (the difficulty in identifying and describing feelings was the mediator). This alternative model showed a good fit, χ^2^(7) = 12.31, *p* = 0.09, CFI = 0.99, TLI = 0.98, RMSEA = 0.03 [90% CI: 0.00, 0.06] similar to the hypothesized mediational model, suggesting that both models could be equally appropriate to explain our data. The second alternative model, in which we posited the difficulty in identifying and describing feelings as the primary predictor, parent and peer attachment as the mediators, and NSSI and suicidal ideation as outcomes, showed a lower fit χ^2^(7) = 30.61, *p* < 0.001, CFI = 0.97, TLI = 0.90, RMSEA = 0.07 [90% CI: 0.05, 0.10] compared to the hypothesized model. Finally, the third alternative model, in which we considered parent and peer attachment as primary predictors, NSSI and suicidal ideation as mediators, and the difficulty in identifying and describing feelings as distal outcome, showed an unacceptable fit χ^2^(7) = 73.74, *p* < 0.001, CFI = 0.92, TLI = 0.70, RMSEA = 0.12 [90% CI: 0.09, 0.14] compared to the hypothesized model.

## Discussion

The primary aim of this study was to investigate the difficulties in identifying and describing feelings, two core facets of alexithymia, and NSSI behavior among a sample of Italian students. Our findings highlighted that self-harmers have higher difficulties in identifying and describing their own feelings, confirming the fact that they are perplexed about their emotions and find it difficult to distinguish between them (Taylor et al., 1997; Hamza et al., 2015). This result moves us in the direction of supporting an affect regulation function of NSSI in which adolescents with difficulties in identifying and describing their feelings may use NSSI as a way of regulating their emotions (Klonsky and Muehlenkamp, 2007), and is consistent with a previous study using a community sample of high school students (Laukkanen et al., 2013).

Importantly, we also tested a theoretical mediational model in which the difficulty in identifying and describing feelings was the mediator of the associations between NSSI and quality of attachment toward both parents and peers. Results supported the hypothesized mediation model: low levels of quality of attachment may enhance the risk of both NSSI and suicidal ideation by compromising adolescents’ abilities to identify their own feelings. This result is in line with findings showing the importance of quality attachment in emotion regulation. Specifically, a negative environment (i.e., neglectful environments) influences the nature and quality of the relationships in which parents and children engage and it may interrupt the development of healthy emotion regulation skills in children and adolescents (Trickett et al., 2011; Peh et al., 2017; Williams et al., 2017). There is evidence that a lack of attachment security in early life affects the development of processes involved in emotion regulation (Schore, 2001; Taylor, 2010). In other words, the child who in absence of a secure attachment to parents fails to develop adequate self-regulatory capacities (Taylor, 2010). Similarly, our results also support the importance in extending the investigation on attachment beyond early childhood through to adolescence, and particularly in investigating the perceptions adolescents have of the quality of their actual attachment relationships. Specifically during adolescence age, the interactions with peers assume an increasingly higher priority, attachment behavior is also often oriented toward non-parental figures (Kerns et al., 2006) because peers are perceived as primary sources of consolation and support.

Accordingly, it seems that poor emotional bonds with both parents and peers may act as distal risk factors for developing later psychopathology. Our findings confirm that NSSI behaviors can be considered as a dysfunctional emotion-regulation strategy in presence of a inability to identifying feelings, consistent with recent results of Peh et al. (2017) that suggested how NSSI may be viewed as maladaptive attempts to cope with negative effects.

However, our model also suggests that these negative effects might be counteracted by helping adolescents understand and recognize their own feelings. In a systematic review of the literature exploring the link between alexithymic features and NSSI, Norman and Borrill (2015) showed that individuals, who are able to understand and communicate their feelings, are likely to engage in NSSI behaviors in order to regulate their emotions. Furthermore, identifying and labeling an emotional experience in itself reduces emotional intensity that, in turn, may help to prevent the perceived need to engage in NSSI (Sleuwaegen et al., 2017).

This result can have high clinical relevance if we consider that, to date, only a few studies have investigated the mediational role played by core alexithymic facets in association with NSSI behaviors and other risk factors during early adolescence and adolescence. For instance, Garisch and Wilson (2010) found that alexithymia mediated the association between bullying and self-harm in high school students. This is in line with the findings of the present study supporting the hypotheses that the inability to regulate and communicate emotions in a normally adaptive way plays an important role in NSSI behaviors. The role of alexithymia in maladaptive behavior was also explored by Swannell et al. (2012) who reported that alexithymia (i.e., difficulty in describing feelings) partially mediated the effect of childhood abuse on self-harm in females but not in males.

Consistent with other studies, our findings indicated that NSSI is moderately prevalent among a non-clinical adolescent population (Hilt et al., 2008). Specifically, 28.8% reported one or more lifetime histories of NSSI behaviors (<5) while 1.4% reported engaging in repetitive NSSI (five or more episodes during the last year) using different methods.

Moreover, adolescents who engaged in NSSI showed a poor perception of quality of attachment with both parents and peers. This is consistent with literature indicating that early attachment relationships have important implications for mental health later in life (Arbuthnott and Lewis, 2015) and in line with study Hallab and Covic’s (2010), which measured attachment by the same tool adopted in this paper and showed that those who self-injured had the worst perceived quality of attachment to parents compared with those who did not intentionally hurt themselves.

With regards to suicidal ideation, findings revealed that suicidal ideation is negatively related to quality of attachment to parents and peers and positively correlated with DIF. Suicidal thought is also positively correlated with the number of stressful events.

Parcel and part of our analysis, we also confirmed that a greater number of life events (as reported by adolescents) were an additional risk factor for NSSI episodes. While a positive perception of the quality of attachment to parents and to peers was related to a smaller number of NSSI behaviors.

However, while a history of childhood trauma has been reported as a common risk factor for NSSI, the role of family, peer relationships and attachment has not been thoroughly explored. Given the limited studies investigating these interrelations, the current study is an important contribution to the extant literature. With regard to the relationship between stressful life events and NSSI, a previous cited study by Paivio and McCulloch (2004) restricted its focus exclusivity on child maltreatment as predictor variable in their mediational model, and did not consider other types of traumatic or stressful life events.

Conversely, consistent with recent research findings (Cerutti et al., 2011; Liu et al., 2014), the present study extend the focus on other important adverse experiences not only maltreatment. In fact, our results provide evidence that an increased frequency of NSSI is associated with a greater number of stressful life events as experience of sexual harassment, being victim of bullying, witnessing potentially traumatic events, etc.

### Limitation

Our study has several limitations that should be addressed. First, our data were correlational in nature and, therefore, no relationships of cause-effect could be established. Second, although we tested for possible alternative models, we recognize that longitudinal data are superior for analyzing mediational hypotheses. In particular, future longitudinal studies should test the possible reciprocal influences among attachment, NSSI, and suicidal ideation as suggested by the good model fit reported by the first alternative model. Third, the value of retrospective histories of stressful or traumatic experiences might be questionable, given the possibility of under-reporting, over-reporting or false memory. Lastly, social desirability response bias may also have affected the results.

### Clinical Implication

Despite these limitations, the present study highlights the risk factors having a significant impact upon NSSI among early adolescents who reported engaging in self-injury behavior when compared to their non-injuring counterparts. These findings add further information to the scant existing literature about possible links between core alexithymic facets (i.e., difficulty in identifying and describing feelings) and NSSI and their relationships with perceived attachment quality, stressful life events and suicidal ideation. Specifically, when the quality of attachment with both peers and parents is compromised, helping children understand their own emotions may reduce the risk of higher NSSI and suicidal thoughts.

However, more research is needed to further explore the variables considered in this study. There is, therefore, a need for more data to better understand this association in order to provide useful information for the planning of preventive interventions in younger populations.

## Author Contributions

All of the authors have substantially and equally contributed to the development and preparation of the manuscript. Furthermore, all the authors have approved the final version of the manuscript. Finally, all authors have agreed to be accountable for all aspects of the manuscript in ensuring that questions related to the accuracy or integrity of any part of it are appropriately investigated and resolved.

## Conflict of Interest Statement

The authors declare that the research was conducted in the absence of any commercial or financial relationships that could be construed as a potential conflict of interest.

## Footnotes

1 Although we also assessed the subdimension of externally oriented thinking, we did not include it because it showed very low reliability (for similar findings and considerations, see also Rieffe et al., 2006).

2 Before computing the reliability the Alienation scale was recoded.
